# Intra-Species Diversity of *Leishmania major* and *L. tropica* from Clinical Isolates of Cutaneous Leishmaniasis in Southwest Iran Inferred by ITS1-rDNA

**Published:** 2019-05

**Authors:** Aliyar MIRZAPOUR, Adel SPOTIN, Hamed BEHNIAFAR, Hakim AZIZI, Bahman MALEKI, Homayon SHAKERAMINIA, Seyyed Javad SEYYED TABAEI

**Affiliations:** 1. Department of Medical Parasitology and Mycology, School of Medicine, Shahid Beheshti University of Medical Sciences, Tehran, Iran; 2. Infectious and Tropical Diseases Research Center, Tabriz University of Medical Sciences, Tabriz, Iran; 3. Student Research Committee, Tabriz University of Medical Sciences, Tabriz, Iran; 4. Department of Medical Parasitology, Zabol University of Medical Sciences, Zabol, Iran; 5. Department of Parasitology, Faculty of Medical Sciences, Tarbiat Modares University, Tehran, Iran; 6. Department of Nursing, School of Nursing, Lorestan University of Medical Sciences, Khoramabad, Iran

**Keywords:** Cutaneous leishmaniasis, Genetic diversity, PCR-RFLP, *Leishmania tropica*, *Leishmania major*, Iran

## Abstract

**Background:**

Cutaneous leishmaniasis (CL) as a public health concern is increasingly circulating by causative agents of *Leishmania tropica* and *L. major* in Iran. As regard to recent treatment failure and controlling problems, the accurate elucidation of heterogeneity traits and taxonomic status of *Leishmania* spp. should be broadly addressed by policymakers. This study was designed to determine the genetic variability and molecular characterization of *L. major* and *L. tropica* from Iranian CL patients.

**Methods:**

One hundred positive Giemsa-stained slides were taken from clinical isolates of CL at Pol-e-Dokhtar County, Southwest Iran, from May 2014 to Sep 2016. DNAs were directly extracted and amplified by targeting ribosomal internal transcribed spacer (ITS) gene following microscopic observation. To identify *Leishmania* spp. amplicons were digested by restriction enzyme *Hae*III subsequent PCR-RFLP technique. To reconfirm, the isolates were directly sequenced to conduct diversity indices and phylogenetic analysis.

**Results:**

Based upon the RFLP patterns, 84 and 16 isolates were explicitly identified to *L. tropica* and *L. major* respectively. No co-infection was found in clinical isolates. The high genetic diversity of *L. tropica* (Haplotype diversity 0.9) was characterized compared to *L. major* isolates (Hd 0.476). The intra-species diversity for *L. tropica* and *L. major* isolates corresponded to 3%–3.9% and 0%–0.4%, respectively.

**Conclusion:**

Findings indicate the *L. tropica* isolates with remarkable heterogeneity than *L. major* are predominantly circulating at Pol-e-Dokhtar County. Occurrence of high genetic variability of *L. tropica* may be noticed in probable treatment failure and/or emerging of new haplotypes; however, more studies are warranted from various geographic regions of Southwest Iran, using large sample size.

## Introduction

Cutaneous leishmaniasis (CL) is a group of neglected vector-borne disease which presents by various clinical appearances and heterogeneity traits (1, 2). CL is dispersed in 20 out of 31 Iranian provinces with overall prevalence of 1.8% to 37.9% and estimated annual incidence of 26630 cases (2–4). In order of clinical-epidemiology importance, two well-known etioparasitological agents, *Leishmania major,* and *L. tropica* are principal causative agents of CL in Iran (3, 5). The genetic diversity of the genus *Leishmania* is discussed as one of the most controversial issues among the endemic areas. The genetic heterogeneity of *Leishmania* parasite may lead to emergence of diverse phenotypic aspects, emergent sub-species/strains, and formation of novel haplotypes, and subsequently rapid generation of drug-resistance alleles (6–9). Currently, *L. major* and *L*. *tropica* are unambiguously circulating in Lorestan province, where it is addressed as a neglected focus of leishmaniasis in Southwest Iran (10).

To evaluate genetic diversity examination of *Leishmania* spp. several nuclear and mitochondrial DNA genes have tested, including Cytochrome b (kDNA maxicircle), microsatellites, gp63, 18S-rRNA, mini-exon, HSP-70 and ribosomal internal transcribed spacer regions (ITS-rDNA) (3, 11–17). The ITS-rDNA region can utilize to understand evolutionary hypotheses of *Leishmania* as a result of low intracellular polymorphism and to be conserved regions (18).

As regard to recent drug resistance and controlling problems, the precise elucidation of single nucleotide polymorphism (SNP) and taxonomic status of *Leishmania* spp. by more sensitive strategies could be generally noticed by regional policymakers. The aim of this study was to determine molecular characterization and genetic variability of *L. major* and *L. tropica* from Pol-e-Dokhtar County, Southwest Iran.

## Methods

### Study area

**Fig. 1:**
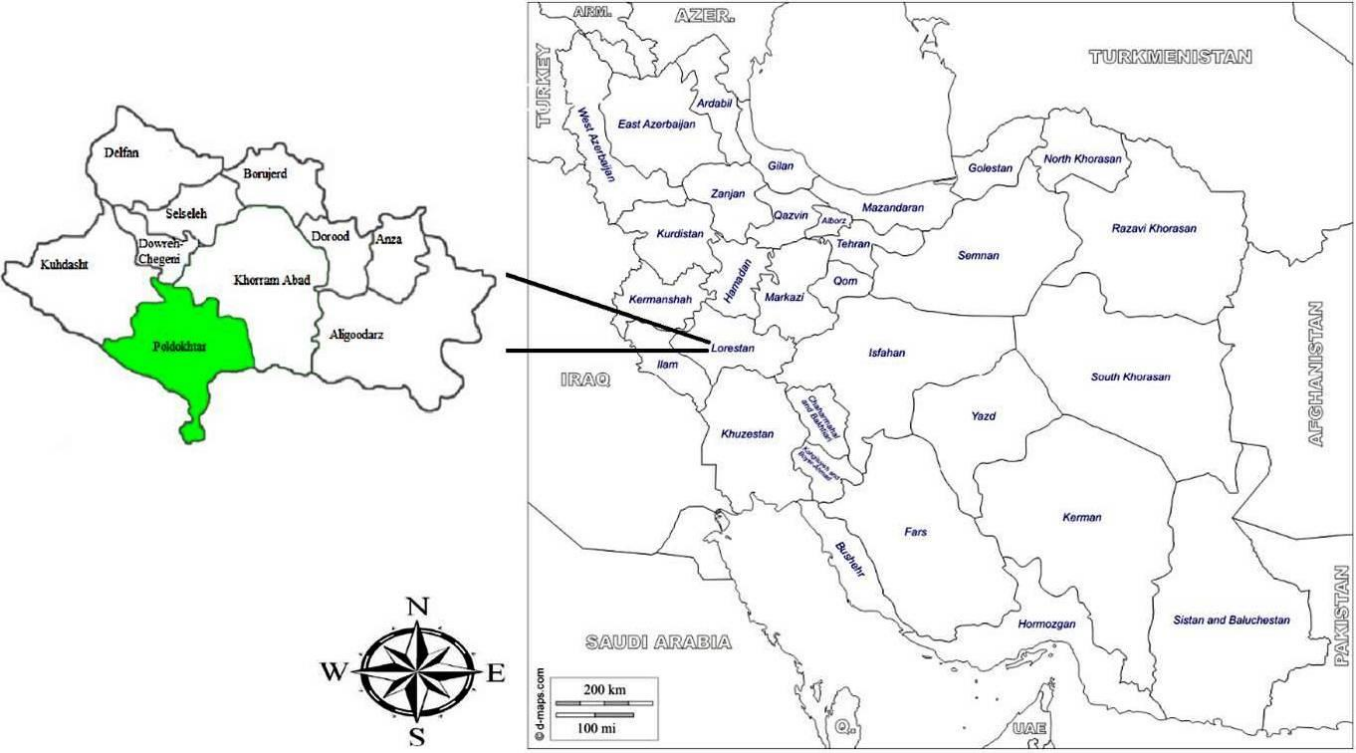
Map of the Lorestan Province and Pol-e-Dokhtar County presenting study locations in Southwest Iran

Pol-e-Dokhtar County is located in the South of the Lorestan Province (Fig. 1). This county occupied 3615 square km, and according to the Statistical Center of Iran (SCI), the population size is 75,327. Pol-e-Dokhtar city has an altitude of 673 meters above sea level. The annual average temperature of the city is 11.9°C.

### Sampling and microscopic examination

The lesions of suspected patients to CL were sampled from Pol-e-Dokhtar Health Center, from May 2014 to Sep 2016. Overall, 100 microscopically confirmed slides were collected. The samples were smeared on microscopic slides, dried, and stained by Giemsa. *Leishmania* infections were identified under light microscope with high magnification (×1000). Demographic and clinical data of each patient including age, sex, locality, the number of lesions, and location of lesion were recorded using a questionnaire.

### Extraction of Total Genomic DNA, PCR amplification and restriction fragment length polymorphism for ITS1-rDNA

DNA of *Leishmania* was directly extracted from positive slides based upon the Phenol-chloroform protocol (6). All Giemsa-stained slides were washed with ethanol and covered with 250*μ*L lysis buffer (50mM NaCl, 50 mMTris, 10mM EDTA, pH 7.4, 1% v/v Triton x-100 and 100 μg of proteinase k per ml). To accomplish, cell lysis samples were then incubated at 56°C for 3 h or overnight. The obtained DNA was re-suspended in 30 μL double-distilled water and stored at −20 °C until molecular doings.

The single round PCR was performed to identify the *Leishmania* infection by targeting ribosomal internal transcribed spacer 1 (ITS1) using previously designed specific primers, LITSR (5′-CTGGATCATTTTCCGATG-3′) as Forward primer and L5.8S (5′TGATACCACTTATCGCACTT-3′) as reverse primer (19). The following program was used to carry out PCR: initial denaturing cycle at 94°C for 5 min, followed by 35 cycles of 94°C for 30 sec, 47 °C for 30 sec, 72 °C for 1 min and finally 1 cycle of 72°C for 5 min. PCR products were run on 1.5%gel agarose stained with ethidium bromide and observed under UV light.

Restriction fragment length polymorphism (RFLP) was performed on PCR products to determine the parasite species. Endonuclease reaction of ITS1-rDNA gene was done in a volume of 30*μ*L containing 2*μ*L of *Bsu*RI (*Hae*III) with cut site GG↓CC, 10*μ*L of PCR products, 2*μ*L of 10× buffer, and 16*μ*L of distilled water for 15 min at 37°C. Finally, the digested fragments were revealed using 3% gel agarose and UV light.

### Sequencing and phylogenetic analyses

To re-confirm the RFLP findings, amplicons were randomly purified and sequenced by targeting ITS1-rDNA gene using ABI 3130X automatic sequencer at the Bioneer Company, South Korea. Contigs (overlapped sequences) were aligned (http://multalin.toulouse.inra.fr/multalin) and edited at consensus positions using Sequencher Tmv.4.1.4 Software (Gene Codes Corporation). The percent identity and divergence (pairwise distances) among sequenced isolates were drawn using the MegAlign program from Laser Gene Bio Computing Software Package (DNASTAR, Madison, WI). To demonstrate the taxonomic status of identified isolates, the phylogenetic tree was constructed by MEGA 5.05 software based on Maximum Likelihood algorithm and kimura two-parameter model. The accuracy of phylogenetic tree was evaluated by 1000 bootstrap re-sampling. The *Trypanosoma brucei* (Accession number: **JN673390**) and *Leishmania mexicana* (Accession number: **AF466383**) were considered as out-group branches. The diversity indices (haplotypes diversity [Hd] and nucleotide diversity [π]) was calculated by DnaSP software version 5.10 (20).

## Results

The demographic and clinical data of CL cases based on sex, age group, and location of lesion are given in Table 1. The vast majority infection was identified in 20–29 yr olds (25%) than other groups and in males (70%). The most lesions located at hand (53%) and foot (26%) (Table 1). The ITS1-rDNA gene (target 361bp) was successfully amplified by single round PCR for all *Leishmania* isolates.

**Table 1:** Frequency of CL cases based on sex, age group and location of lesion in Poldokhtar County, Iran in 2014 to 2016

***Variable***		***%***
Sex	Male	70
	Female	30
Age group (according to years)	<9	13
	0–19	21
	20–29	25
	30–39	14
	40–49	15
	50≤	12
Location of lesion	Hand	53
	Foot	26
	Face	13
	Hand and foot	2
	Hand and face	5
	Hand, foot and face	1

**Fig. 2:**
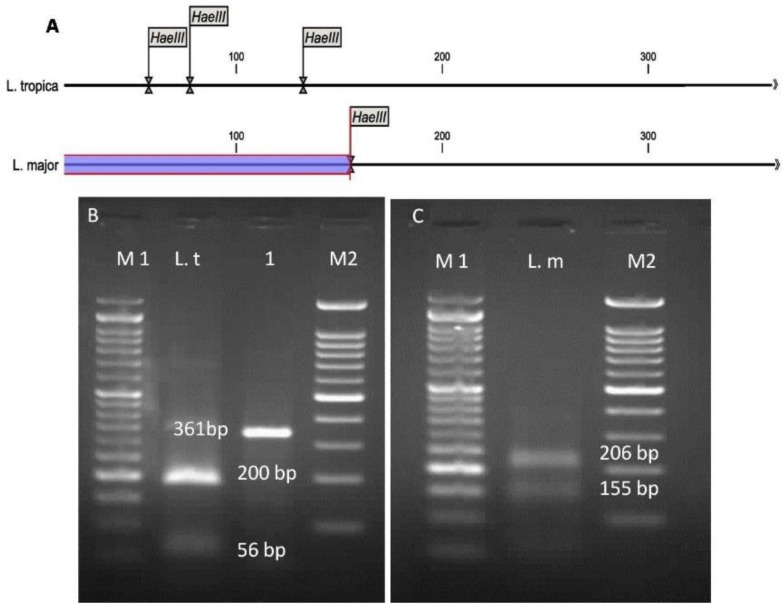
A: *In-silico* prediction of *Hae*III restriction enzyme for ITS1-rDNA gene in the *L. major* and *L. tropica*. The digested fragments due to PCR-RFLP process in Pol-e Dokhtar County based on ITS1-rDNA gene. B: M1: 50bp ladder marker, L. t: *L. tropica* (fragments 57bp, 56bp, and 200bp), Lane 1: The amplified *Leishmania* spp. in infected patients (361 bp), M2: 100 bp ladder marker. C: L. m: *L. major (*fragments 155 bp and 206 bp)

**Fig. 3:**
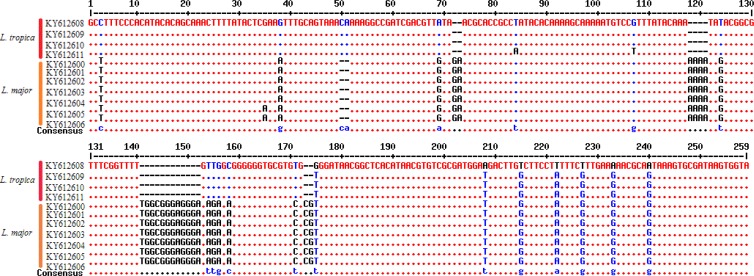
The multiple sequence alignment of ITS1-rDNA gene for *L .major* and *L. tropica*

**Fig. 4:**
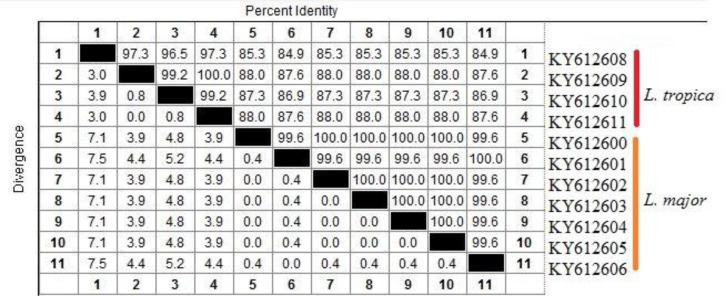
The intra/inter species identity/diversity between *L .major* and *L. tropica* identified in this study

**Fig. 5:**
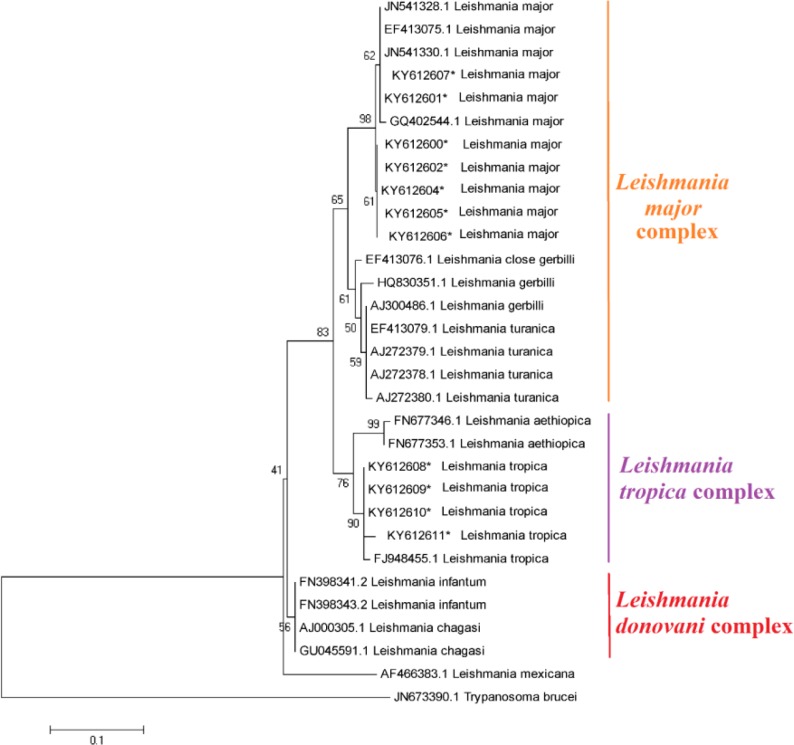
Maximum Likelihood bootstrap tree indicating the relationships of the haplotypes of ITS1-rDNA gene for *L .major* and *L. tropica*. Only bootstrap values of higher than 60% are indicated on each branch. The *Trypanosoma brucei* (Accession number: **JN673390**) and *Leishmania mexicana* (Accession number: **AF466383**) were considered as out-group branches. In this study, the registered sequences marked by asterisk (*)

The *in-silico* prediction of *Bsu*R1 restriction enzyme for ITS1-rDNA gene of *L .major* and *L. tropica* is shown in Fig. 2A. Based upon the RFLP digested patterns, 84 and 16 isolates were identified to *L. tropica* (fragments 57bp, 56bp and 200bp) and *L. major* (fragments 155bp and 206bp) isolates, respectively (Fig. 2 B–C). No co-infection was found in identified isolates. The high genetic diversity of *L. tropica* (Hd 0.9) was characterized compared to *L. major* (Hd 0.476). *L. tropica* sequences (0.02176) had more nucleotide diversity than *L. major* isolates (0.00183) (Table 2). The multiple sequence alignment of ITS1-rDNA gene for *L .major* and *L. tropica* is shown in Fig. 3. The intra-species diversity and identity for *L. tropica* isolates were 3–3.9% and 96–97.3% while, for *L. major* isolates corresponded to 0%–0.4% and 99.6%, respectively (Fig. 4). The topology of constructed phylogenetic tree disclosed that the *L. major* (Accession nos: **KY612600**-**KY612607**) and *L. tropica* (Accession nos: **KY612608**-**KY612611**) were grouped with bootstrap value of higher than 60% in their specific complex (Fig. 5). The *Trypanosoma brucei* and *Leishmania mexicana* were considered as out-group branches.

**Table 2:** Diversity and neutrality indices of *Leishmania major* and *Leishmania tropica* based on nucleotide sequences of internal transcribed spacer 1–rDNA gene in Pol-e-Dokhtar County, Lorestan Province, Iran N: number of isolates; Hn: number of haplotypes; Hd: haplotype diversity; Nd: nucleotide diversity.

***Parasite***	***N***	***Hn***	***Hd± SD***	***Nd (π) ± SD***
*L. major*	10	2	0.476±0.171	0.00183±0.00157
*L. tropica*	10	4	0.900±0.02592	0.02176±0.00088

## Discussion

In this investigation, different levels of genetic diversity of *L. tropica* and *L. major* isolates were identified in clinical isolates of CL patients from Southwest Iran, inferred by ITS1-rDNA nucleus gene.

*L. tropica* (n=86%) was firmly determined by RFLP and phylogenetic analyses as the principal species responsible for CL in the Pol-e-Dokhtar County, where here was not an eligible information concerning taxonomic status and heterogeneity traits of parasite causing CL (10). Pol-e-Dokhtar County is known as a neglected hyperendemic focus of leishmanisis, which neighbored with Khuzestan and Ilam provinces. Current finding shows that the most CL infections were occurred in males with age group 20–29 yr old. This may be males frequently work in the field places and females cover their body and apply Hijab because of religion (3).

In this study, nuclear ribosomal internal transcribed spacer region as a universal DNA barcode marker was used to identify probable haplotypes and evolutionary relationship of *L. tropica* and *L. major* because of its conserve features and be multi-copy (18).

In the current findings, the *L. tropica* isolates demonstrated a greater genetic diversity (Hd: 0.9 and nucleotide diversity; 0.02176) than *L. major* (hd: 0.476 and nucleotide diversity: 0.00183). This may be described by the genomic characters of ITS-rDNA. GC content of *L. tropica* is generally lower than *L. major* (59.7%), therefore the stability of triple hydrogen bonds of the GC pair and stacking interaction is faced with a slippage (21, 22). Furthermore, this heterogeneity may elucidate by several facts; First: presence of turnover mechanisms; unequal crossing over/transposition and slippage in the sequence length of parasite(23). Second: lack of any bottleneck effects.

One of the limitations of current investigation is that the study area was highly confined and the isolates are relatively small to be inferred for more extensive larger-scale genetic diversities, However; we cannot explicitly estimate the heterogeneity levels of *L. tropica* and *L. major* in the region. On the one hand, the employing evolutionary mitogenome markers (e.g. *Cytochrome* b or *Cyt* c oxidase subunit I) can identify the new haplotypes than nuclear genes (e.g., ITS1-rDNA).

Up to now, the genetic diversity of *Leishmania* spp. have been reported by several regional researchers by targeting nucleus (ITS-rDNA) and/or mitochondrial (kDNA and *Cy*t b) markers from various endemic foci of Iran.

The sequence alignment of kDNA gene demonstrated a high genetic diversity of *L. major* from different rural and urban areas of Fars Province (Southern Iran) (24).

However, by targeting ITS-rDNA gene a high genetic polymorphism of *L. major* (7.3%) than *L. tropica* isolates (3.6%) had shown in various locations of Iran (25), whilst we contrary found more variations in *L. tropica* to be compared to *L. major* isolates.

A high genetic diversity of *L. tropica* compared to *L. major* isolates by targeting both ITS-rDNA and *Cyt* b genes had shown from Southwestern Iran (Khuzestan Province) (3).

In addition, by using kDNA, CL causing strains of *L. major* in southern Iran have a significant genetic variation (26).

Tashakori et al. have demonstrated the genetic heterogeneity of Iranian isolates of *L. major* using single-strand conformation polymorphism and sequence analysis of the ribosomal ITS (27).

In another study, genetically high polymorphism of *L. major* isolates from CL patients was demonstrated in Central Iran (Isfahan Province) (28).

The considerable genetic diversity of *L. major* in RNA polymerase II largest subunit (RPOIILS) and lack of the genetic diversity in mitochondrial DNA polymerase beta (DPOLB) were reported in Central Iran (Isfahan Province) (29).The genetic diversity in *L. major* strains was determined belong to different endemic areas of Iran using kDNA, there was considerable diversity between strains of different regions and even between isolates that belong to the same area (30).

This study revealed that both of main causative agents of CL are present in Pol-e-Dokhtar County. In accordance with previous studies, *L. tropica*was found predominant species in the study area (10, 31). *L. tropica*and *L. major* were the main causative agents of CL in Pol-e-Dokhtar County with the occurrence of 82.2 and 35.3% respectively (10, 31).

In this study, the most affected part of the body was belonged to hand, foot, and face, respectively. This finding is in accordance with the study conducted in the same area (31).

## Conclusion

The *L. tropica* isolates with remarkable heterogeneity than *L. major* are predominantly circulating at Pol-e-Dokhtar County. Occurrence of high genetic variability of *L. tropica* may be noticed in probable treatment failure and/or emerging of new haplotypes; however, more studies are warranted from various geographic regions of Southwest Iran, using large sample size.

## Ethical considerations

Ethical issues (Including plagiarism, informed consent, misconduct, data fabrication and/or falsification, double publication and/or submission, redundancy, etc.) have been completely observed by the authors.
